# High-Frequency, High-Throughput Quantification of SARS-CoV-2 RNA in Wastewater Settled Solids at Eight Publicly Owned Treatment Works in Northern California Shows Strong Association with COVID-19 Incidence

**DOI:** 10.1128/mSystems.00829-21

**Published:** 2021-09-14

**Authors:** Marlene K. Wolfe, Aaron Topol, Alisha Knudson, Adrian Simpson, Bradley White, Duc J. Vugia, Alexander T. Yu, Linlin Li, Michael Balliet, Pamela Stoddard, George S. Han, Krista R. Wigginton, Alexandria B. Boehm

**Affiliations:** a Department of Civil & Environmental Engineering, Stanford University, Stanford, California, USA; b Verily Life Sciences, South San Francisco, California, USA; c California Department of Public Health, Infectious Diseases Branch, Richmond, California, USA; d County of Santa Clara Public Health Department, San Jose, California, USA; e County of Santa Clara Department of Environmental Health, San Jose, California, USA; f Department of Civil & Environmental Engineering, University of Michigan, Ann Arbor, Michigan, USA; UCSF

**Keywords:** COVID-19, SARS-CoV-2, settled solids, wastewater

## Abstract

A number of recent retrospective studies have demonstrated that severe acute respiratory syndrome coronavirus 2 (SARS-CoV-2) RNA concentrations in wastewater are associated with coronavirus disease 2019 (COVID-19) cases in the corresponding sewersheds. Implementing high-resolution, prospective efforts across multiple plants depends on sensitive measurements that are representative of COVID-19 cases, scalable for high-throughput analysis, and comparable across laboratories. We conducted a prospective study across eight publicly owned treatment works (POTWs). A focus on SARS-CoV-2 RNA in solids enabled us to scale up our measurements with a commercial lab partner. Samples were collected daily, and results were posted to a website within 24 h. SARS-CoV-2 RNA in daily samples correlated with the incidence of COVID-19 cases in the sewersheds; a 1 log_10_ increase in SARS-CoV-2 RNA in settled solids corresponds to a 0.58 log_10_ (4×) increase in sewershed incidence rate. SARS-CoV-2 RNA signals measured with the commercial laboratory partner were comparable across plants and comparable to measurements conducted in a university laboratory when normalized by pepper mild mottle virus (PMMoV) RNA. Results suggest that SARS-CoV-2 RNA should be detectable in settled solids for COVID-19 incidence rates of >1/100,000 (range, 0.8 to 2.3 cases per 100,000). These sensitive, representative, scalable, and comparable methods will be valuable for future efforts to scale up wastewater-based epidemiology.

**IMPORTANCE** Access to reliable, rapid monitoring data is critical to guide response to an infectious disease outbreak. For pathogens that are shed in feces or urine, monitoring wastewater can provide a cost-effective snapshot of transmission in an entire community via a single sample. In order for a method to be useful for ongoing COVID-19 monitoring, it should be sensitive for detection of low concentrations of SARS-CoV-2, representative of incidence rates in the community, scalable to generate data quickly, and comparable across laboratories. This paper presents a method utilizing wastewater solids to meet these goals, producing measurements of SARS-CoV-2 RNA strongly associated with COVID-19 cases in the sewershed of a publicly owned treatment work. Results, provided within 24 h, can be used to detect incidence rates as low as approximately 1/100,000 cases and can be normalized for comparison across locations generating data using different methods.

## INTRODUCTION

The coronavirus disease 2019 (COVID-19) pandemic has prompted the rapid and widespread maturation of wastewater-based epidemiology (WBE). Through the first year of the pandemic, retrospective analyses of wastewater samples for severe acute respiratory syndrome coronavirus 2 (SARS-CoV-2) RNA demonstrated strong correlations between SARS-CoV-2 target concentrations and infection incidence (1–8). Wastewater monitoring can therefore provide a cost-effective snapshot of transmission in a community by using just one sample to provide information that is unbiased by access to testing or symptom status. To be of most value for public health officials, the methods used to produce data should be (i) sensitive to detect low levels of viral RNA in wastewater, (ii) representative of the disease rates in the community, (iii) scalable to provide high-throughput results with a short turnaround time, and (iv) comparable between labs and different approaches.

Numerous independent COVID-19 WBE efforts have covered monitoring at a range of scales, from the building level to country-wide implementation (7, 9, 10). The methods employed have also varied, with the vast majority focusing on the liquid fraction of municipal wastewater. Due to the dilute nature of SARS-CoV-2 RNA in wastewater, most methods that focus on influent require a number of preanalytical steps to concentrate SARS-CoV-2 prior to extracting the viral RNA. Depending on the method, these steps include ultrafiltration (2, 5), organic flocculation coupled to centrifugation (3, 11), and charged membrane filtration (12). Although liquid wastewater monitoring can be utilized for large programs, each of these listed concentration steps add significant time, equipment, and personnel requirements to SARS-CoV-2 RNA quantification in wastewater. For effective monitoring, methods need to be scalable. SARS-CoV-2 RNA measurements should be prospective as opposed to retrospective, should provide high-resolution (e.g., daily) data on SARS-CoV-2 levels, and should result in data within hours or days of sample collection. As wastewater monitoring expands to more communities, methods focused on quantifying SARS-CoV-2 in liquid are not readily amenable to scale up and automation due to the additional steps needed to prepare the samples.

The majority of SARS-CoV-2 RNA in wastewater originates in the feces of infected individuals. Even after mixing with wastewater liquid, coronaviruses have a stronger affinity to the solid fraction of wastewater, even higher than the affinities of nonenveloped viruses (13). Studies comparing SARS-CoV-2 RNA concentrations in the liquid and solid fractions of real wastewater have demonstrated that the solids harbor 3 to 4 orders of magnitude higher concentrations of SARS-CoV-2 RNA on a per mass basis than liquid influent (3, 14). As a result, the settling of wastewater solids that happens during primary treatment at most wastewater treatment plants serves as a built-in concentration step for wastewater monitoring. SARS-CoV-2 RNA quantification from these samples simultaneously requires less preanalytical processing (limited to centrifugation and resuspension of solids) and, on a per mass basis, will result in higher measured concentrations than measurements made on the liquid fraction of wastewater despite the potential challenge of inhibitory compounds that are present in solids. If plants do not have a primary clarifier or samples are taken at the subsewershed level, solids may still be concentrated from influent using standard methods (15). Thus, sampling is quite flexible, and samples may be obtained as grab samples from a primary clarifier, manually composited from primary clarifier samples, or settled out from grab or composite liquid samples. SARS-CoV-2 monitoring focused on settled solids may therefore be both more sensitive and more conducive to scale up and automation.

In this study, we demonstrate that a SARS-CoV-2 monitoring project focused on wastewater solids can be readily scaled to produce sensitive results that are representative of COVID-19 incidence through a high-frequency effort conducted through a commercial laboratory with <24-h turnaround times. We initiated a prospective monitoring project across eight POTWs in the greater San Francisco Bay and Sacramento areas of California to measure SARS-CoV-2 RNA in daily samples. Results were consistently reported to stakeholders and agencies within 24 h of sample collection. Here we show that SARS-CoV-2 RNA in daily samples correlates strongly with COVID-19 incidence rates in the sewersheds. Results indicate SARS-CoV-2 RNA signals are comparable across plants and across laboratories using different measurement methods. We present the empirical sensitivity of the measurements to incidence rates in the sewersheds.

## RESULTS

### Quality assurance (QA)/quality control (QC).

Negative and positive extraction and PCR controls were negative and positive, respectively. Bovine coronavirus (BCoV) recoveries were, on average 57% (standard deviation = 39%), and all were above 1% (see Fig. S1 in the supplemental material), indicating lack of substantial inhibition and good recovery of viral RNA during extraction. Sample standard deviations for the SARS-CoV-2, pepper mild mottle virus (PMMoV), and BCoV recovery quantification estimated from the merged wells were, on average 19%, 19%, and 14% of the measurement, respectively. As the samples were extracted 10 times and each extract was analyzed in 1 of 10 replicate wells which were merged, the replicate variability incorporates variation from both RNA extraction and reverse transcription-digital droplet PCR (RT-ddPCR) with a heterogeneous solid sample. Additional reporting according to dMIQE guidelines and wastewater data used in this study are available through the Stanford Digital Repository (16).

10.1128/mSystems.00829-21.2 FIG S1 Distribution of BCoV recovery at each POTW. The line through the box represents the median, and the top and bottom of the box represent the 75th and 25th percentiles, respectively. The top and bottom whiskers show 1.5 times the upper and lower interquartile range, respectively. Data are shown as light gray symbols. Download FIG S1, TIF file, 0.9 MB. Copyright © 2021 Wolfe et al. 2021 Wolfe et al. https://creativecommons.org/licenses/by/4.0/ This content is distributed under the terms of the Creative Commons Attribution 4.0 International license.

### POTW adherence to protocols.

Adherence to daily sampling was high among publicly owned treatment works (POTWs). During the duration of this study, POTW staff were unable to collect samples on 0 day (4 POTW), 1 day (2 POTW), 2 days (1 POTW), and 10 days (1 POTW). The reason for missed samples was most often lack of sampling bottles or miscommunication with new staff. For the POTW that missed 10 samples, samples were missed mostly on Sundays and holidays when the POTW had limited staff on site.

### Measurement overview.

Across all samples, PMMoV ranged from 4.3 × 10^7^ to 7.1 × 10^9^ copies (cp)/g (average = 6.6 × 10^8^). PMMoV was different between POTW (Kruskal-Wallis *P* < 10^−15^) with Ocean tending to have the lowest PMMoV and Gil the highest (Fig. S2). SARS-CoV-2 RNA gene concentrations ranged from 630 to 3.7 × 10^6^ (N gene), nondetected (ND) to 3.2 × 10^6^ (S gene), and ND to 3.0 × 10^6^ (ORF1a) cp/g across all samples. A total of four samples returned nondetects during this period (one for S and three for ORF1a), and the value of 300 cp/g (approximately half the detection limit) was substituted for these for further analysis. N, S, and ORF1a gene concentrations were highly correlated (correlation coefficient [*r_p_*] ranged from 0.97 to 0.98 for log_10_-transformed data aggregated across plants). Pairwise linear regressions between log_10_-transformed N, S, and ORF1a concentrations returned slopes of ∼1 (Fig. S3). Results with untransformed variables were similar (see supplemental material). Therefore, further analyses in this paper focus on the N gene alone.

10.1128/mSystems.00829-21.3 FIG S2 Distribution of log_10_ PMMoV at each POTW. The line through the box represents the median, and the top and bottom of the box represent the 75th and 25th percentiles, respectively. The top and bottom whiskers show 1.5 times the upper and lower interquartile range, respectively. Data are shown as light gray symbols. Download FIG S2, TIF file, 0.9 MB. Copyright © 2021 Wolfe et al. 2021 Wolfe et al. https://creativecommons.org/licenses/by/4.0/ This content is distributed under the terms of the Creative Commons Attribution 4.0 International license.

10.1128/mSystems.00829-21.4 FIG S3 Relationship between different SARS-CoV-2 RNA targets across all samples. Slopes of linear regression are provided along with standard error of regression coefficient for the lines shown. Slopes of regressions of raw data (not log_10_ transformed) are 1.0 (N versus S), 1.1 (N versus ORF1a), and 1.1 (S versus ORF1a), and correlation coefficients (*r_p_*) between the same variables are 0.97, 0.97, and 0.99, respectively. Download FIG S3, TIF file, 0.8 MB. Copyright © 2021 Wolfe et al. 2021 Wolfe et al. https://creativecommons.org/licenses/by/4.0/ This content is distributed under the terms of the Creative Commons Attribution 4.0 International license.

### Relationship between SARS-CoV-2 RNA in solids and incident cases.

**FIG 1:**
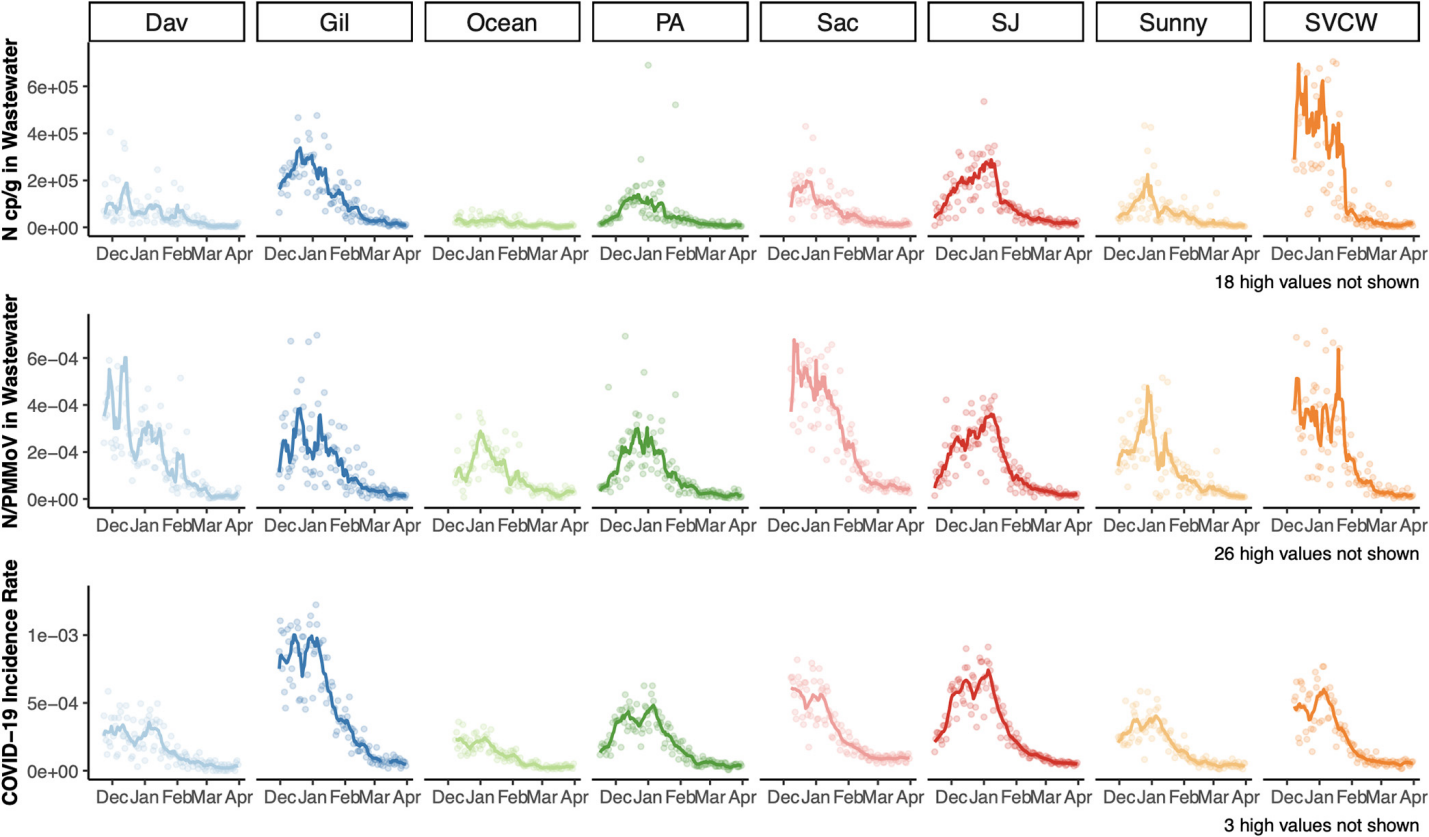
Time series of (top to bottom) N copies per gram (cp/g) in wastewater, N/PMMoV, and laboratory-confirmed SARS-CoV-2 incidence rate (bottom) for each of eight POTWs and their sewersheds from mid-November/December 2020 to 31 March 2021. Points represent daily values. Lines are 7-day, centered smoothed averages. For wastewater, this is a trimmed average where the highest and lowest values from the seven day period are removed before averaging.

Concentrations of SARS-CoV-2 RNA rose over the “winter surge” in COVID-19 incidence in November and December of 2020 and declined to lower levels at the end of March (Fig. 1). There is an apparent dip in incidence rate at the peak of the surge; this was probably due to decreased test seeking behavior and reduced testing availability during the week between Christmas and New Year’s Day. During the time period of this study, there were no days on which any POTW achieved a nondetect across the three genes, nor were there days where incident case numbers in the sewershed were 0. Over the duration of the study, the lowest smoothed incident rates observed across the eight POTWs ranged from 1.9 to 8.5 cases per 100,000 people (at Dav and Sac, respectively). These correspond to the low numbers of daily smoothed incident cases ranging from 1.3 to 125.7 in these two sewersheds. Wastewater data availability preceded case data availability; case reporting delays in this region at the time were greater than 3 days.

Kendall’s tau between 7-day smoothed incidence rates and N gene concentrations ranged from 0.46 (Ocean) to 0.70 (SJ) (all *P* < 0.001) (Table 1; see also Fig. S4 to S6 in the supplemental material). Within POTWs, associations were significantly enhanced when N was normalized by PMMoV or scaled by *F* at five of the eight POTWs, although the effect size was small (effect size < 0.1, Kruskal Wallis *P* < 0.001); at Gil, Sun, and SVCW, the association was significantly weakened (effect size < 0.1, Kruskal Wallis *P* < 0.001). When 7-day trimmed average N gene concentrations were used in lieu of raw N gene concentrations, the significance and direction of differences in association between N, N normalized by PMMoV, and N scaled by *F* and incidence rate were unchanged at each plant. Kendall’s tau was significantly higher for each of these measurements when data were trimmed, although the effect size was small (effect size < 0.1, Kruskal Wallis all *P* < 10^−15^).

**TABLE 1:** Empirical Kendall’s tau for the test of the null hypothesis that the wastewater variable is not associated with the 7-day smoothed new COVID-19 incidence rate in the sewershed the day of the wastewater measurement^*a*^

Plant	Median Kendall’s tau					
	N cp/g		N/PMMoV		F*N	
	Daily	Trimmed	Daily	Trimmed	Daily	Trimmed
All	0.657	0.684	0.585	0.601	0.586	0.601
SJ	0.699	0.743	0.709	0.739	0.718	0.740
PA	0.632	0.650	0.648	0.680	0.648	0.674
Sun	0.565	0.618	0.560	0.618	0.557	0.612
Gil	0.697	0.721	0.575	0.646	0.578	0.648
SVCW	0.643	0.660	0.608	0.553	0.605	0.553
Ocean	0.456	0.477	0.546	0.510	0.530	0.499
Dav	0.637	0.662	0.640	0.664	0.641	0.665
Sac	0.595	0.605	0.641	0.657	0.640	0.656

a Values are shown for each sewershed for both daily values and 7-day trimmed wastewater values for N copies/gram (cp/g) and N normalized by PMMoV. Empirical *P* < 0.001 for all.

10.1128/mSystems.00829-21.5 FIG S4 Distribution of Kendall’s tau for each POTW, testing the null hypothesis of no association between wastewater measurements and SARS-CoV-2 incidence. N = 1,000 for each box. The line through the box represents the median, and the top and bottom of the box represent the 75th and 25th percentiles, respectively. The top and bottom whiskers show 1.5 times the upper and lower interquartile range, respectively, and symbols show data points beyond 1.5 times the interquartile range. For all POTWs and wastewater scales, *P* < 0.001. Download FIG S4, JPG file, 0.2 MB. Copyright © 2021 Wolfe et al. 2021 Wolfe et al. https://creativecommons.org/licenses/by/4.0/ This content is distributed under the terms of the Creative Commons Attribution 4.0 International license.

**FIG 2:**
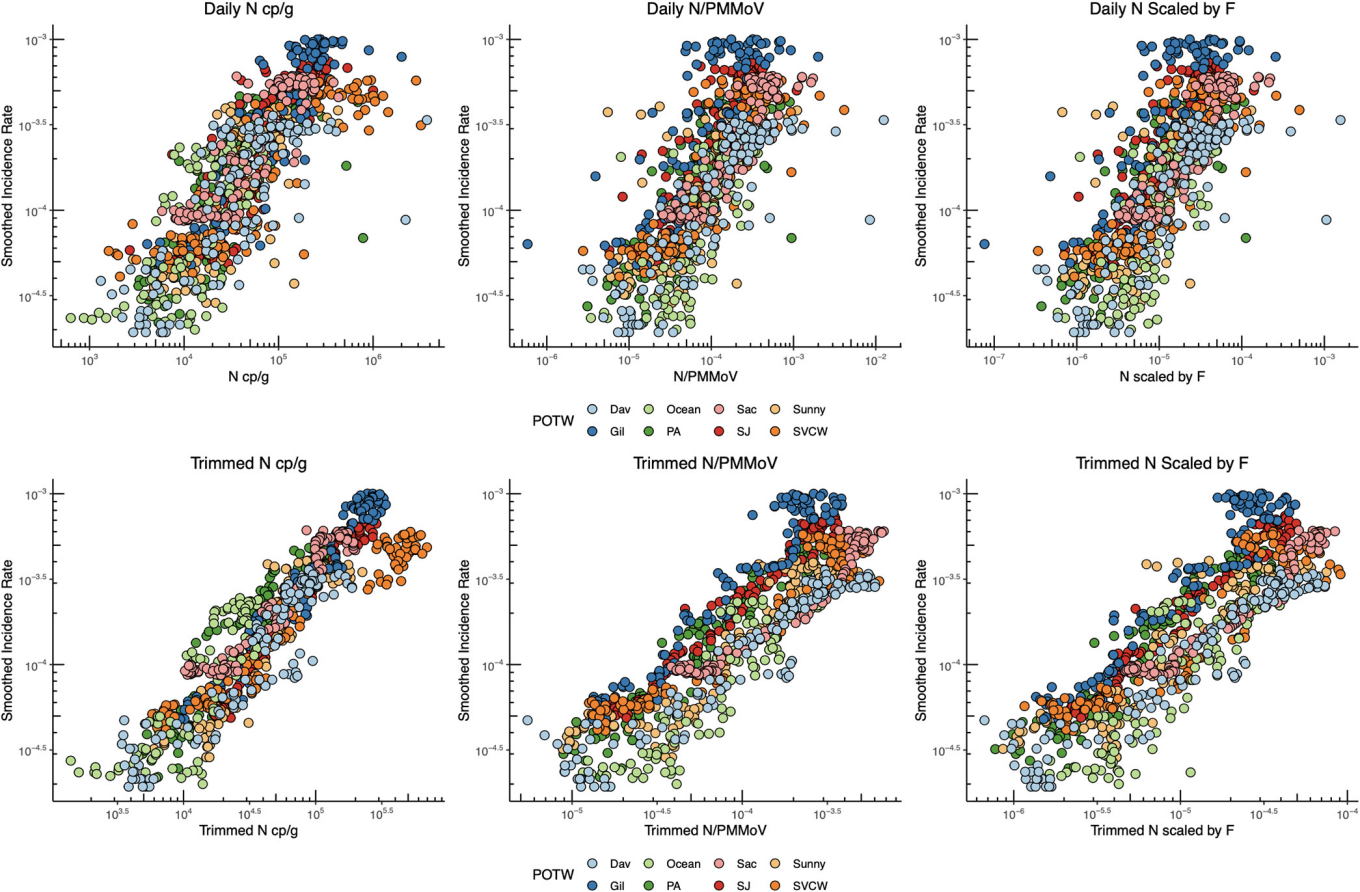
7-day smoothed COVID-19 incidence rate plotted against daily wastewater measurements (top row) and 7-day trimmed wastewater measurements (bottom row). From left to right, plots show the association between incidence rate and N gene copies (gc)/g, N gc/g normalized by PMMoV, and N gc/g scaled by a factor (*F*) that includes PMMoV, TSS, and partitioning coefficients presented in Wolfe et al. (7).

When data from the eight POTWs are aggregated, a strong association between wastewater concentrations of SARS-CoV-2 RNA and incidence rate persists (Fig. 2). Kendall’s tau between raw N concentrations and incidence rates aggregated across plants is 0.66 (*P* < 0.001); tau decreases to 0.58 (*P* < 10^−15^) when data are normalized by PMMoV or scaled by *F* (Table 1 and Fig. S7). Associations between incidence rate and 7-day trimmed average wastewater data are similar to those between incidence rate and raw wastewater data (Table 1). Linear regressions between COVID-19 incidence rate and wastewater values suggest that for a 1 log_10_ increase in N cp/g, there is a 0.59 (± 0.01 standard error) log_10_ increase in incidence rate (*R*^2^ = 0.67), and for N normalized by PMMoV and N scaled by F, there is a 0.58 (±0.02) log_10_ increase in COVID-19 incidence (*R*^2^ = 0.58).

10.1128/mSystems.00829-21.6 FIG S5 7-day smoothed COVID-19 incidence rate plotted against daily wastewater measurements by POTW. From top to bottom, plots show the association between incidence rate and N gene copies/g, N gc/g normalized by PMMoV, and N gc/g s scaled by a factor (F) that includes PMMoV, TSS and partitioning coefficients presented in Wolfe et al. (7). Download FIG S5, JPG file, 0.4 MB. Copyright © 2021 Wolfe et al. 2021 Wolfe et al. https://creativecommons.org/licenses/by/4.0/ This content is distributed under the terms of the Creative Commons Attribution 4.0 International license.

10.1128/mSystems.00829-21.7 FIG S6 7-day smoothed COVID-19 incidence rate plotted against 7-day trimmed wastewater measurements by POTW. From top to bottom, plots show the association between incidence rate and N gene copies/g, N gc/g normalized by PMMoV, and N gc/g s scaled by a factor (F) that includes PMMoV, TSS, and partitioning coefficients presented in Wolfe et al. (7). Download FIG S6, JPG file, 0.3 MB. Copyright © 2021 Wolfe et al. 2021 Wolfe et al. https://creativecommons.org/licenses/by/4.0/ This content is distributed under the terms of the Creative Commons Attribution 4.0 International license.

10.1128/mSystems.00829-21.8 FIG S7 Distribution of Kendall’s tau for data from all POTWs combined, testing the null hypothesis of no association between wastewater measurements and SARS-CoV-2 incidence. N = 1,000 for each box. The line through the box represents the median, and the top and bottom of the box represent the 75th and 25th percentiles, respectively. The top and bottom whiskers show 1.5 times the upper and lower interquartile range, respectively, and symbols show data points beyond 1.5 times the interquartile range. For all POTWs and wastewater scales, *P* < 0.001. Download FIG S7, JPG file, 0.09 MB. Copyright © 2021 Wolfe et al. 2021 Wolfe et al. https://creativecommons.org/licenses/by/4.0/ This content is distributed under the terms of the Creative Commons Attribution 4.0 International license.

**FIG 3:**
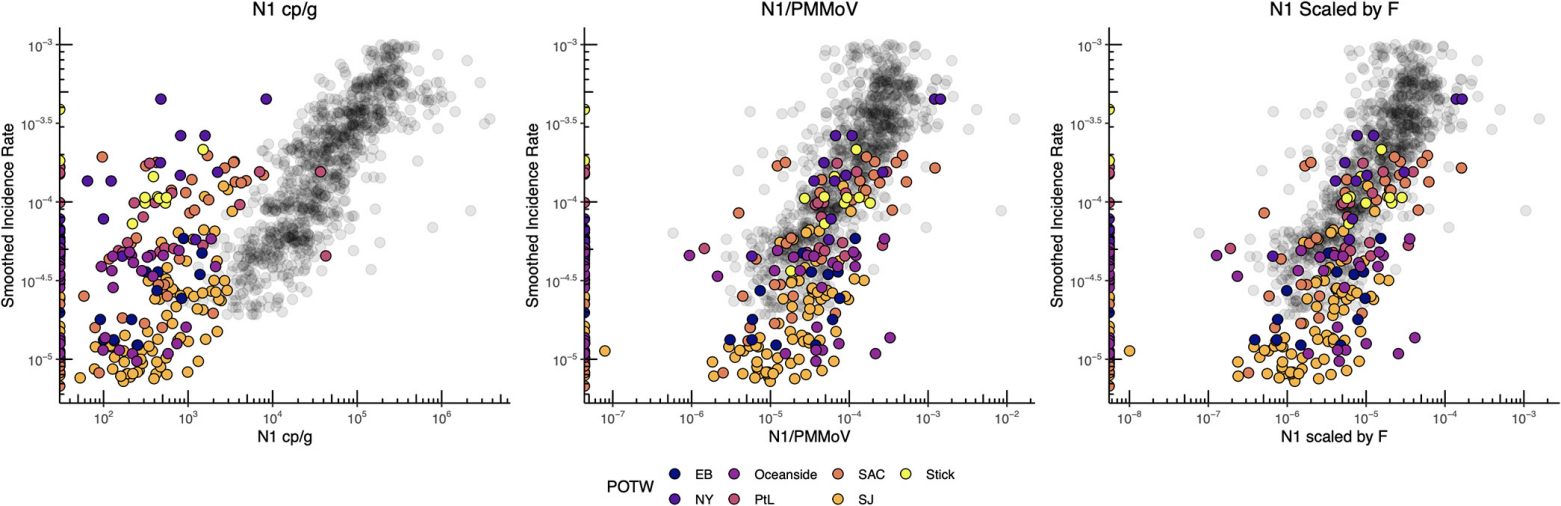
Data from Wolfe et al. (7) (in color) showing daily measurement of SARS-CoV-2 in wastewater overlaid on results from Fig. 2 showing daily measurements from this study. Both data sets show 7-day smoothed COVID-19 incidence rate plotted against daily wastewater measurements of N gene copies/g, N gc/g normalized by PMMoV, and N gc/g scaled by *F*. The two data sets are both generated from analysis of solids; however, Wolfe et al. (7) used a more labor-intensive, small scale set of methods and slightly different genomic targets versus the high-throughput methods presented in this paper.

Incidence rates and concentrations of the N gene in settled solids reported by Wolfe et al. (7) were added to the aggregated plots (Fig. 3). Those data were generated in a different laboratory using a different method from those used for the data reported herein and represent data from diverse POTWs in California, Illinois, and New York during a different phase of the pandemic (spring-fall 2020). When displayed as raw N gene concentrations versus incidence rate, the Wolfe et al. data do not fall on the same data cloud as the data generated in the present study, and the slopes of the linear regression lines through each data set are divergent (slope = 0.13 ± 0.03 log_10_ incidence rate/log_10_ N cp/g for Wolfe et al. [7] and 0.59 ± 0.01 log_10_ incidence rate/log_10_ N cp/g for the data from this paper). However, when all the data are normalized by PMMoV or scaled by a factor that includes total suspended solids (TSS) and partitioning coefficients, the Wolfe et al. data collapse onto the data generated in this study. After normalizing by PMMoV or scaling by F, the regression lines for the two methods remain significantly different (Kruskal Wallis *P* < 0.001 for all); however, the slopes describing the relationship between wastewater values and incidence rate are similar between the two methods after applying these approaches (slope = 0.24 ± 0.03 and 0.58 ± 0.02 log_10_ incidence rate/log_10_ wastewater value for both normalized and scaled data from Wolfe et al. [7] and this paper, respectively).

### Empirical detection limits.

The linear relationship between log_10_-transformed incidence rate and log_10_-transformed N cp/g for each plant was used to estimate the incidence rate detection limit, assuming an assay detection limit of 750 cp/g. The minimum number of estimated cases detectable in each sewershed was calculated based on the population served by each POTW. Across the eight POTWs, the average estimated incidence rate detection limit was 1.4 cases per 100,000 people (range, 0.8 to 2.3 cases per 100,000 depending on POTW; Table 2). In the POTW service areas included in this project, these rates correspond to between 1.0 and 26.3 cases per sewershed depending on sewershed (Dav and Sac are represented by the minimum and maximum report in the range, respectively). This estimation is corroborated by measurement of N gene at concentrations above 750 cp/g when there were 1.3 recorded cases at Dav, although data in this study do not reach the detection limit and future data can lend more insight to these predictions.

**TABLE 2:** Coefficients from bootstrapped regression models describing the relationship between log_10_-transformed COVID-19 incidence and log_10_-transformed N cp/g for each plant and the lowest estimated incidence rate and number of cases expected to result in positive wastewater result

Plant	Intcpt^*a*^	Slope	*R* ^2^	Population	Lowest estimated incidence rate (no. of cases/100,000)^*b*^	Lowest estimated no. of cases^*b*^	Range of observed incidence rate (no. of cases/100,000)	Range of observed no. of cases
SJ	−7.21	0.74	0.75	1,458,017	0.8 (0.8–0.8)	11.8 (11.5–12.2)	4.9–74.2	71.1–1,081.7
PA	−6.66	0.63	0.62	213,968	1.4 (1.3–1.4)	2.9 (2.9–3.0)	2.2–48.3	4.7–103.4
Sun	−6.73	0.62	0.61	169,000	1.1 (1.1–1.2)	1.9 (1.9–2.0)	2.9–40.5	4.9–68.4
Gil	−7.12	0.74	0.80	110,338	1.0 (1.0–1.0)	1.1 (1.0–1.1)	4.8–100.2	5.3–110.6
SVCW	−5.91	0.44	0.75	220,000	2.3 (2.3–2.3)	5.0 (5.0–5.1)	4.1–60.1	9.0–132.1
Ocean	−6.46	0.58	0.46	250,000	1.6 (1.5–1.7)	4.0 (3.9–4.2)	2.0–24.6	5.0–61.4
Dav	−6.31	0.52	0.61	66,622	1.6 (1.5–1.6)	1.0 (1.0–1.1)	1.9–35.8	1.3–23.9
Sac	−6.50	0.61	0.76	1,480,000	1.8 (1.7–1.8)	26.3 (25.6–27.0)	8.6–61.1	127.4–904.0

^*a*^Intcpt is the intercept.

^*b*^Estimated incidence rate and incidence are shown as the median predicted value from bootstrapping with the 25th and 75th percentiles in parentheses. The observed range of 7-day smoothed incidence rate and number of cases during the study period is also shown.

## DISCUSSION

Outbreak monitoring through clinical data alone can present challenges—for diseases where individuals may be infectious before onset of symptoms or asymptomatic such as COVID-19 and norovirus, among others, tests may not be sought or may be unavailable. During the COVID-19 pandemic, racial and ethnic minority groups in the United States have been at higher risk of morbidity and mortality in part due to lack of access to testing and care (17). Wastewater monitoring for SARS-CoV-2 RNA can provide an estimate of COVID-19 incidence rates in communities that are unbiased by these factors. For example, apparent dips in incidence rates associated with testing bias during the holidays were not reflected in wastewater trends. We modified an academic laboratory protocol for measuring SARS-CoV-2 RNA in wastewater solids so that it could be executed quickly (in less than 24 h) and with numerous samples in parallel. Using the high-throughput and rapid protocol, we provided daily measurements of SARS-CoV-2 RNA and associated quality assurance control metrics to stakeholders on a daily basis via a public website (wbe.stanford.edu). Over the period of more than 4 months represented in this analysis, sample results were always available within 24 h of sample retrieval from the POTW with the exception of holidays. POTW staff rarely missed sample collection.

The strong association between SARS-CoV-2 RNA in solids and incident case rates within sewersheds and across sewersheds is striking. The nearly linear relationships between these measures, whether they are examined for individual POTWs or data aggregated across all POTWs, provide evidence that measurements of SARS-CoV-2 RNA in wastewater solids reflect trends in COVID-19 incidence rates for the sewershed. Importantly, associations are strong for all POTWs, and there is no difference between plants that provided grab samples of settled solids, composite samples of settled solids, or solids settled from composite influent samples. This suggests that even for plants without a primary clarifier, solids represent a viable matrix for wastewater surveillance efforts. While 750 cp/g is the estimated detection limit for the methods used for this data, the detection limit can be lowered even further with some adjustments to these methods.

As evidenced by the strong relationship between POTW-aggregated SARS-CoV-2 RNA gene concentrations and incidence rate, adjusting for POTW-specific characteristics (like fecal strength, flow rate, TSS, for example) is not necessary to arrive at an empirical relation between the two variables for the POTWs included in this study. While removing outliers from the wastewater data using a trimmed average approach improves the visual association between wastewater measurements and incidence rates, the statistical measures of association (Kendall’s tau) do not differ substantially between trimmed averages and are used in lieu of raw gene concentrations. Similarly, normalizing by PMMoV or scaling the SARS-CoV-2 RNA concentrations by a factor accounting for PMMoV, TSS, and virus partitioning (*F*) does not improve the strength of the associations. Because TSS is so similar across POTWs (Table S2) and the same partitioning coefficients were used in calculating *F* for each POTW, it is not surprising that the scaling N gene concentrations by *F* does not appreciably affect associations with incidence rate; this was also reported by Wolfe et al. (7).

10.1128/mSystems.00829-21.10 TABLE S2 Summary statistics of total suspended solids (TSS) in the influent wastestreams of the 8 POTWs. Units are milligrams per liter. Average (ave), maximum (max), minimum (min), median (med), and standard deviation (stdev) over the days samples were analyzed are provided. Download Table S2, DOCX file, 0.01 MB. Copyright © 2021 Wolfe et al. 2021 Wolfe et al. https://creativecommons.org/licenses/by/4.0/ This content is distributed under the terms of the Creative Commons Attribution 4.0 International license.

We suggest that SARS-CoV-2 RNA gene concentrations should be normalized by PMMoV and a trimmed average applied for stakeholder and public consumption of the data. Normalizing by PMMoV serves a number of purposes. (i) It adjusts for variable viral RNA recovery between samples (assuming PMMoV RNA recovery is similar to that of SARS-CoV-2 RNA). (ii) It adjusts for differences in fecal strength of the wastestream. (iii) The normalization falls from a mass balance model relating the number of shedders to concentrations in wastewater solids (7). Although normalizing by PMMoV did not improve the associations observed in data from this study, the benefit of this approach is especially realized in our analysis, illustrating that results from two distinct laboratories using distinct methods to measure SARS-CoV-2 RNA in solids can be combined when SARS-CoV-2 RNA concentrations are normalized by PMMoV. In this case, the median recovery of BCoV spiked into the solids described by Wolfe et al. (7) was 4%, while BCoV recovery in the present study was approximately 10 times higher. As such, normalizing by PMMoV likely served to adjust for variable recovery. Additionally, recent work by Simpson et al. (18) suggests that normalizing by PMMoV can serve to correct for degradation of SARS-CoV-2 RNA during sample storage.

A benefit of having daily measurements is the ability to apply a trimmed averaging approach for data visualization. We recommend applying a trimmed average to eliminate the influence of outliers during data visualization by public health professionals and nonexperts, including the public. Environmental data exhibit variability that is caused by different factors than those that are most familiar to these audiences, e.g., biases that cause variability in clinical case or syndromic data. SARS-CoV-2 RNA concentrations in wastewater may exhibit high-frequency variability for several reasons. Among the most influential factors are the following: (i) changing contributions to the sample from sudden movement of people shedding viral RNA in their stool into or out of the sewershed or intermittent deliveries of septic waste that could vary in content or time represented relative to wastewater flows, (ii) variability in fecal shedding rates from person to person (19, 20), and (iii) samples are spatially heterogeneous, and although our samples are well mixed, inhomogeneities could exist at spatial scales greater than those captured by our sampling.

### Conclusions.

We measured three SARS-CoV-2 RNA targets in wastewater solids daily for over 4 months for consumption by POTWs and public health stakeholders. The methods used were shown to be (i) sensitive to identify low concentrations of SARS-CoV-2 and COVID-19 incidence rates in the associated sewersheds, (ii) scalable to a high-throughput format with results delivered daily within 24 h, (iii) representative of disease incidence in the sewersheds served, and (iv) comparable across laboratories using different methods to analyze solids. POTW staff provided samples and rarely missed a sample. Using a high-throughput method that takes advantage of automation and robotics, we were able to provide sample results within 24 h of sample receipt and displayed those results on a public website for stakeholders. As the use of wastewater monitoring is poised to scale globally for monitoring not just COVID-19, but also other diseases, it is critical that methods are scalable for use by laboratories producing reliable results on an industrial scale. Strict QA/QC procedures (including negative and positive extraction and PCR controls for all targets, and recovery controls) coupled to replicate analyses (*n* = 10) for each sample ensured high-quality data. Measurements at each POTW are strongly associated with laboratory-confirmed COVID-19 incidence rates in the sewersheds dated to the time of specimen collection. Further, the strong association persists when data are aggregated across POTWs, suggesting that SARS-CoV-2 RNA concentrations in settled solids from different POTWs can be directly compared to infer relative incidence rates across POTW sewersheds. Although normalizing data by PMMoV was not necessary for comparing measurements made at different POTWs in this study, we show how normalizing by PMMoV can allow for data from wastewater solids measured by different methods and laboratories to be readily compared to each other. Public health representatives have expressed an interest in comparing these data across sewersheds. Future work will utilize a longer time series of daily wastewater measurements from these POTWs to investigate the appropriate cadence of sampling to capture incident case trends during different phases of the pandemic and utilize solids settled from samples capturing subsewershed areas to illustrate use of these methods at different scales.

## MATERIALS AND METHODS

A wastewater monitoring program was initiated in November 2020 to implement methods previously developed for analysis of wastewater solids (3) in a high-throughput format for high-resolution (daily) data collection at eight publicly owned treatment works (POTWs) in the greater San Francisco Bay and Sacramento areas of California. Whereas the original protocol took several days to complete, the high-throughput protocol uses liquid-handling robots and other automations to process samples in less than 24 h. These improvements are primarily due to changes in preanalytical processing and RNA extraction, including the resuspension of solids in a buffer and the use of automated extraction protocols. Protocols used for sample processing as described below are publicly available on protocols.io (21–23), and results of wastewater measurements have been provided to stakeholders in real time on a website visualizing the data (wbe.stanford.edu). Four POTWs serve populations of Santa Clara County, CA (SJ, Gil, Sun, and PA), and the others each serve a portion of San Mateo County (SVCW and PA), San Francisco County (Ocean), Sacramento County (Sac), and Yolo County (Dav). The eight POTWs serve between 66,000 and 1,500,000 residents and have permitted flows between 8.5 and 181 million gallons per day (Table 3).

**TABLE 3:** Description of POTWs and samples in this study^*a*^

Acronym	County	Population served	Permitted flow (MGD)	Pretreatment?	Solids collected from	Residence time (h)^*b*^ in:		Grab/composite?	Start date (*n*)^*c*^
						Clarifier	Sewer network		
SJ	Santa Clara	1,458,017	167	FeCl_3_	Primary clarifier	1–2	4–18	C	15 Nov (137)
PA	Santa Clara	213,968	39		Primary clarifier	2–3	2–9	G	16 Nov (135)
Sun	Santa Clara	169,000	29.5		Primary clarifier	3–6	6–12	G	30 Nov (122)
Gil	Santa Clara	110,338	8.5		Influent	NA	3–6	C	30 Nov (121)
SVCW	San Mateo	220,000	29	FeCl_3_	Primary clarifier	2–3	2–8	G	8 Dec (114)
Ocean	San Francisco	250,000	43		Primary clarifier	3–6	6–12	G	8 Dec (104)
Dav	Yolo	66,622	7.5		Primary clarifier	2	2	G	23 Nov (127)
Sac	Sacramento	1,480,000	181		Primary clarifier	1	15	G	8 Dec (114)

a The study started in 2020.

b Information on the residence time of sewage in the network and solids in the clarifier was obtained through a survey completed by POTW operators and managers.

c *n* in the last column is the number of samples analyzed at each POTW. Nov, November; Dec, December.

### Sample collection.

Samples were collected by POTW staff using sterile technique in clean, labeled bottles provided by our team. POTWs were not provided additional compensation for participation. Approximately 50 ml of settled solids was collected each day from each sewage treatment plant between mid-to-late November 2020 and 31 March 2021. At seven of the eight POTWs, settled solids were collected from the primary clarifier; at these POTWs, the residence time of solids in the primary clarifier ranged from 1 to 6 h (Table 3). Settled solids samples were grab samples at all plants except for SJ. At SJ, POTW staff manually collected a 24-h composite sample (3) (Table 3). At Gil, solids were settled from a 24-h composite influent sample using standard method 160.5 (15). Samples were immediately stored at 4°C and transported to the commercial partner laboratory by a courier service where processing began within 6 h of collection.

### Sample preparation.

The solids were dewatered by centrifugation at 24,000 × *g* for 30 min at 4°C. The supernatant was aspirated and discarded. A 0.5- to 1-g aliquot of the dewatered solids was dried at 110°C for 19 to 24 h to determine its dry weight. Bovine coronavirus (BCoV) was used as a positive recovery control. Each day attenuated bovine coronavirus vaccine (PBS Animal Health, Calf-Guard Cattle Vaccine) was spiked into DNA/RNA shield solution (Zymo Research) at a concentration of 1.5 μl/ml. Dewatered solids were resuspended in the BCoV-spiked DNA/RNA shield to a concentration of 75 mg/ml. This concentration of solids was chosen as previous work titrated solutions with various concentrations of solids to identify a concentration that minimized inhibition while maintaining sensitivity of SARS-CoV-2 assays. High recoveries of spiked BCoV in samples (see Results) indicates lack of significant inhibition in the samples at this concentration of solids. Between 5 and 10 5/32-in. Stainless steel grinding balls (OPS Diagnostics) were added to each sample which was subsequently homogenized by shaking with a Geno/Grinder 2010 (Spex SamplePrep). Samples were then briefly centrifuged to remove air bubbles introduced during the homogenization process and then vortexed to remix the sample.

### RNA extraction.

RNA was extracted from 10 replicate aliquots per sample. For each replicate, RNA was extracted from 300 μl of homogenized sample using the Chemagic Viral DNA/RNA 300 kit H96 for the Perkin Elmer Chemagic 360 followed by PCR inhibitor removal with the Zymo OneStep-96 PCR Inhibitor Removal kit. Extraction-negative controls (water) and extraction-positive controls were extracted using the same protocol as the homogenized samples. The positive controls consisted of 500 copies of SARS-CoV-2 genomic RNA (ATCC VR-1986D) in the BCoV-spiked DNA/RNA shield solution described above.

### Droplet digital PCR.

RNA extracts were used as the template in digital droplet RT-PCR assays for SARS-CoV-2 N, S, and ORF1a RNA gene targets in a triplex assay and BCoV and PMMoV in a duplex assay (see Table S1 for primer and probe sequences, purchased from IDT). During the study period, new SARS-CoV-2 variants emerged—three targets were used to increase confidence in the performance of these measurements as the virus changes over time, and the conserved regions targeted did not contain known mutations in common variants. PMMoV is highly abundant in human stool and domestic wastewater globally (24, 25) and is used here as an internal recovery and fecal strength control. Undiluted extract was used for the SARS-CoV-2 assay template, and a 1:100 dilution of the extract was used for the BCoV/PMMoV assay template. Digital RT-PCR was performed on 20-μl samples from a 22-μl reaction volume, prepared using 5.5-μl template, mixed with 5.5 μl of One-Step RT-ddPCR Advanced kit for Probes (catalog no. 1863021; Bio-Rad), 2.2 μl reverse transcriptase, 1.1 μl dithiothreitol (DTT), and primers and probes at a final concentration of 900 nM and 250 nM, respectively. Droplets were generated using the AutoDG Automated Droplet Generator (Bio-Rad). PCR was performed using Mastercycler Pro with the following protocol: reverse transcription at 50°C for 60 min, enzyme activation at 95°C for 5 min, 40 cycles with 1 cycle consisting of denaturation at 95°C for 30 s and annealing and extension at either 59°C (for SARS-CoV-2 assay) or 56°C (for PMMoV/BCoV duplex assay) for 30 s, enzyme deactivation at 98°C for 10 min, and then an indefinite hold at 4°C. The ramp rate for temperature changes was set at 2°C/s, and the final hold at 4°C was performed for a minimum of 30 min to allow the droplets to stabilize. Droplets were analyzed using the QX200 Droplet Reader (Bio-Rad). All liquid transfers were performed using the Agilent Bravo (Agilent Technologies).

10.1128/mSystems.00829-21.9 TABLE S1 The molecular targets used in this study as well as the primer and probe sequences. The N, S, and ORF1a genes are located within the SARS-CoV-2 genome. The BCoV target is for bovine coronavirus, a process control spiked into the sample during processing. PMMoV is for the internal endogenous control which is naturally present in high concentrations in the samples. Additional details of these assays can be found in Huisman et al. (28). Download Table S1, DOCX file, 0.01 MB. Copyright © 2021 Wolfe et al. 2021 Wolfe et al. https://creativecommons.org/licenses/by/4.0/ This content is distributed under the terms of the Creative Commons Attribution 4.0 International license.

Each sample was run in 10 replicate wells, extraction-negative controls were run in 7 wells, and extraction-positive controls in 1 well. In addition, PCR-positive controls for SARS-CoV-2 RNA, BCoV, and PMMoV were run in 1 well, and no-template controls (NTC) were run in 7 wells. Positive controls consisted of BCoV and PMMoV gene block controls (double-stranded DNA [dsDNA] purchased from IDT) and guide RNA (gRNA) of SARS-CoV-2 (ATCC VR-1986D). Results from replicate wells were merged for analysis. Thresholding was done using QuantaSoft Analysis Pro software (Bio-Rad, version 1.0.596). Additional details are provided in supporting material per the dMIQE guidelines (26). In order for a sample to be recorded as positive, it had to have at least three positive droplets. Three positive droplets corresponds to a concentration between ∼500 and 1000 copies (cp)/g; the range in values is a result of the range in the equivalent mass of dry solids added to the wells.

Concentrations of RNA targets were converted to concentrations per dry weight of solids in units of copies/gram (dry weight) using dimensional analysis. The total error is reported as standard deviations and includes the errors associated with the Poisson distribution and the variability among the 10 replicates. The recovery of BCoV was determined by normalizing the concentration of BCoV by the expected concentration given the value measured in the spiked DNA/RNA shield. BCoV recovery was used solely as a process control and not used in the calculation of concentrations; samples were rerun in cases where the recovery of BCoV was less than 1%. Data were provided daily after quality control checks by the commercial laboratory partner and data management and analysis led by Stanford University.

### (i) Ancillary wastewater data.

Wastestream influent total suspended solid (TSS) concentrations in milligram/liter data were obtained from POTW staff. If TSS measurements were not coincident on the day that a sample was taken, the TSS value for that day was estimated using linear interpolation.

### (ii) COVID-19 case data.

Counts of laboratory-confirmed COVID-19 incident cases as a function of episode date (earliest of reported symptom onset, laboratory result, or case record create dates) for each sewershed were obtained from local or state sources through data-use agreements. Case data were aggregated within the sewersheds based on georeferenced reported home addresses, which were delineated using the POTW-specific geographic information system (GIS) shape files. COVID-19 incidence rates per 100,000 population were calculated using the estimated population served in each sewershed.

### Statistical analysis.

Statistics were computed using RStudio (version 1.1.1073). For ddRT-PCR data, results are reported both daily and using a 7-day, centered trimmed moving average where the largest and smallest values are dropped prior to averaging. COVID-19 incidence data are represented in terms of 7-day, centered running average (smoothed) new cases for each of the sewersheds. The latter is justified owing to the “weekend effect” associated with a reduction in test seeking behavior, testing availability, and result reporting (27). Hereafter, any reference to incidence or incidence rate will refer to the value from the 7-day smoothed average.

Incidence rates were compared to SARS-CoV-2 RNA concentrations, SARS-CoV-2 RNA concentrations normalized by PMMoV concentrations (*C*_PMMoV_), and SARS-CoV-2 RNA concentrations scaled by the factor *F* = *K*_dp_(1 + *K_d_*TSS)/[C_PMMoV_*K_d_*(1 + *K*_dp_TSS)] where *K_d_* and *K*_dp_ are the partitioning coefficients for SARS-CoV-2 and PMMoV, respectively, and the other terms have been defined. The scaling factor falls from a mass balance model relating the number of SARS-CoV-2 fecal shedders in a sewershed to the concentration of SARS-CoV-2 RNA in settled solids (7) and will hereafter be referred to as *F* for simplicity. In applying *F* to the data, we used *K_d_* = 1,000 and *K*_dp_ = 100 (7). Results were similar when the analysis was repeated with various *K_d_* and *K*_dp_ between 100 and 10^4^ ml/g (data not shown).

Nonparametric Kendall’s tau and Kruskal-Wallis methods were used to test hypotheses regarding associations and trends as data were neither normally nor log normally distributed based on Shapiro-Wilk tests. Linear regressions were used to assess slopes describing relationships between incidence rates and N gene concentrations normalized by PMMoV for each POTW and for all POTWs aggregated. To account for variability of wastewater measurements, Kendall’s tau empirical *P* values and regression coefficients *m* were determined using 1,000 bootstrap resamplings that incorporate nondetect measurements and measurement errors (3), and median tau, regression coefficients *m*, standard error, *R*^2^, and empirical *P* values are reported. For trimmed data, bootstrapping utilized 95% confidence intervals accounting for variability in the five measurements included in the trimmed average.

The minimum incidence rate at which wastewater solids contain measurable SARS-CoV-2 RNA was estimated for each POTW. Predictions were calculated within the bootstrapping approach by using coefficients from the observed linear relationship between log_10_-transformed COVID-19 incidence rate and log_10_ N cp/g measured in wastewater solids for each bootstrapped sample to predict the incidence rate when wastewater solids contained 750 cp/g of the N gene. The median predicted incidence rate and interquartile range were reported. The 750 cp/g value was chosen because it falls in the middle of the range of the lowest detectable concentration of the method.

Concentrations of SARS-CoV-2 N1 and PMMoV targets in settled solids reported by Wolfe et al. (7) collected early in the pandemic (spring-fall 2020) at seven POTWs, including two from this project (SJ and Ocean) were acquired along with associated sewershed COVID-19 incidence rates. These data were used to assess whether trends observed with data in the present study are distinct from those reported by Wolfe et al. (7). The empirical regression coefficients for the two data sets were compared using the Kruskal-Wallis test to test the null hypothesis of no difference between the distribution of coefficients. Those authors measured N1 and PMMoV using a different workflow than used in the present study. The detection limit of N1 reported by those authors was ∼40 cp/g; in assessing associations using these data, the bootstrapping approach samples from a uniform distribution defined by 0 and 40 cp/g to assign a value to nondetects in the data set.

10.1128/mSystems.00829-21.1 TEXT S1 Bootstrapping code. Bootstrapping was used to determine Kendall’s tau empirical *P* values and regression coefficients *m*. The code utilized 1,000 bootstrap resamplings that incorporate measurement errors and replaced nondetect values with the range below the limit of detection (LOD). A snippet of R code that shows an example of how the sampling was done for bootstrapping is shown. Download Text S1, DOCX file, 0.01 MB. Copyright © 2021 Wolfe et al. 2021 Wolfe et al. https://creativecommons.org/licenses/by/4.0/ This content is distributed under the terms of the Creative Commons Attribution 4.0 International license.
