# Supplementary figures and images for: High-Frequency, High-Throughput Quantification of SARS-CoV-2 RNA in Wastewater Settled Solids at Eight Publicly Owned Treatment Works in Northern California Shows Strong Association with COVID-19 Incidence
Source: mSystems. 2021 Sep 14;6(5):e00829-21. doi: 10.1128/mSystems.00829-21 (PMC8547422; doi:10.1128/mSystems.00829-21)

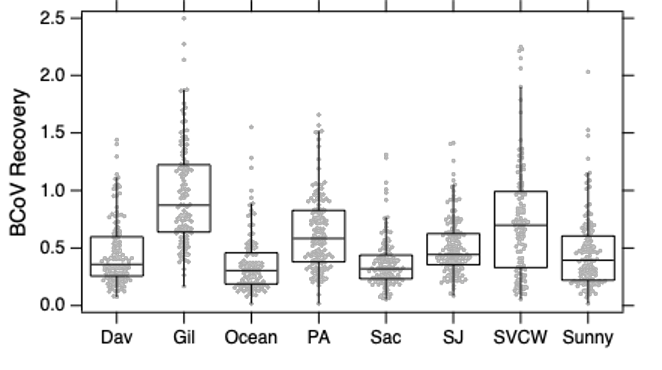

Supplement: FIG S1 [file msystems.00829-21-sf001.tif]

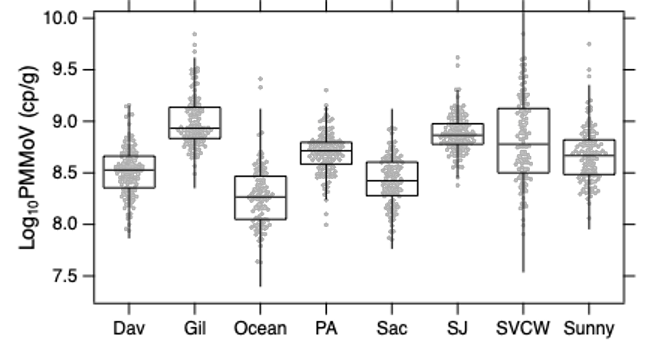

Supplement: FIG S2 [file msystems.00829-21-sf002.tif]

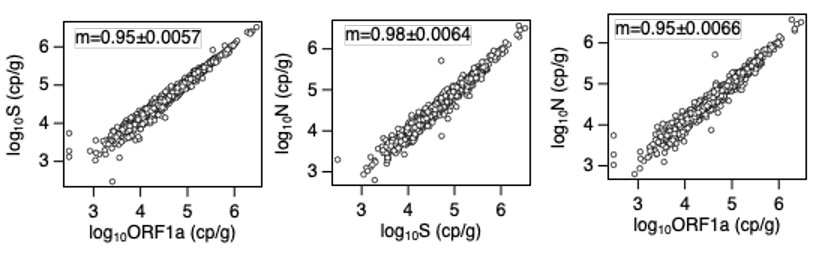

Supplement: FIG S3 [file msystems.00829-21-sf003.tif]

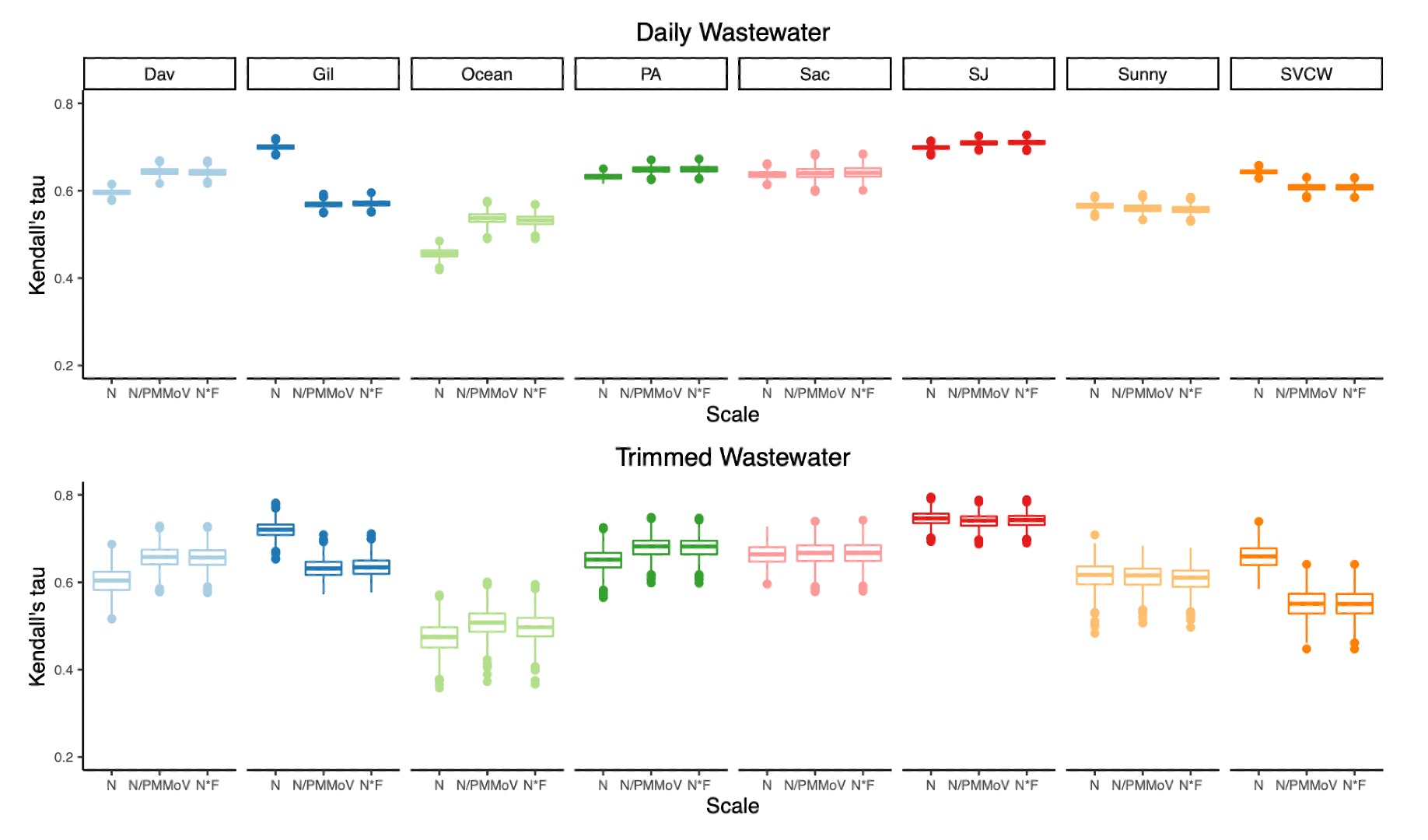

Supplement: FIG S4 [file msystems.00829-21-sf004.jpg]

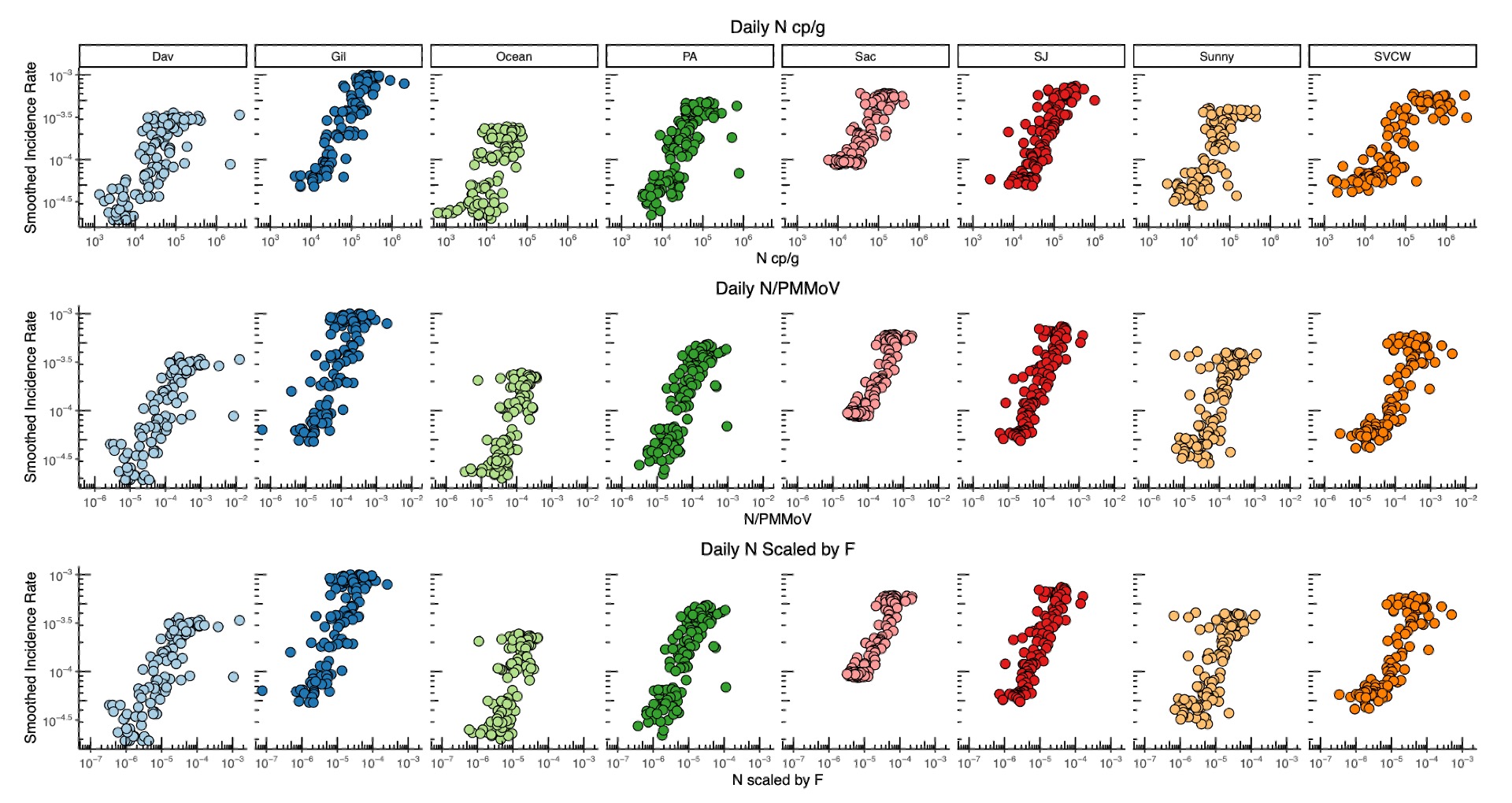

Supplement: FIG S5 [file msystems.00829-21-sf005.jpg]

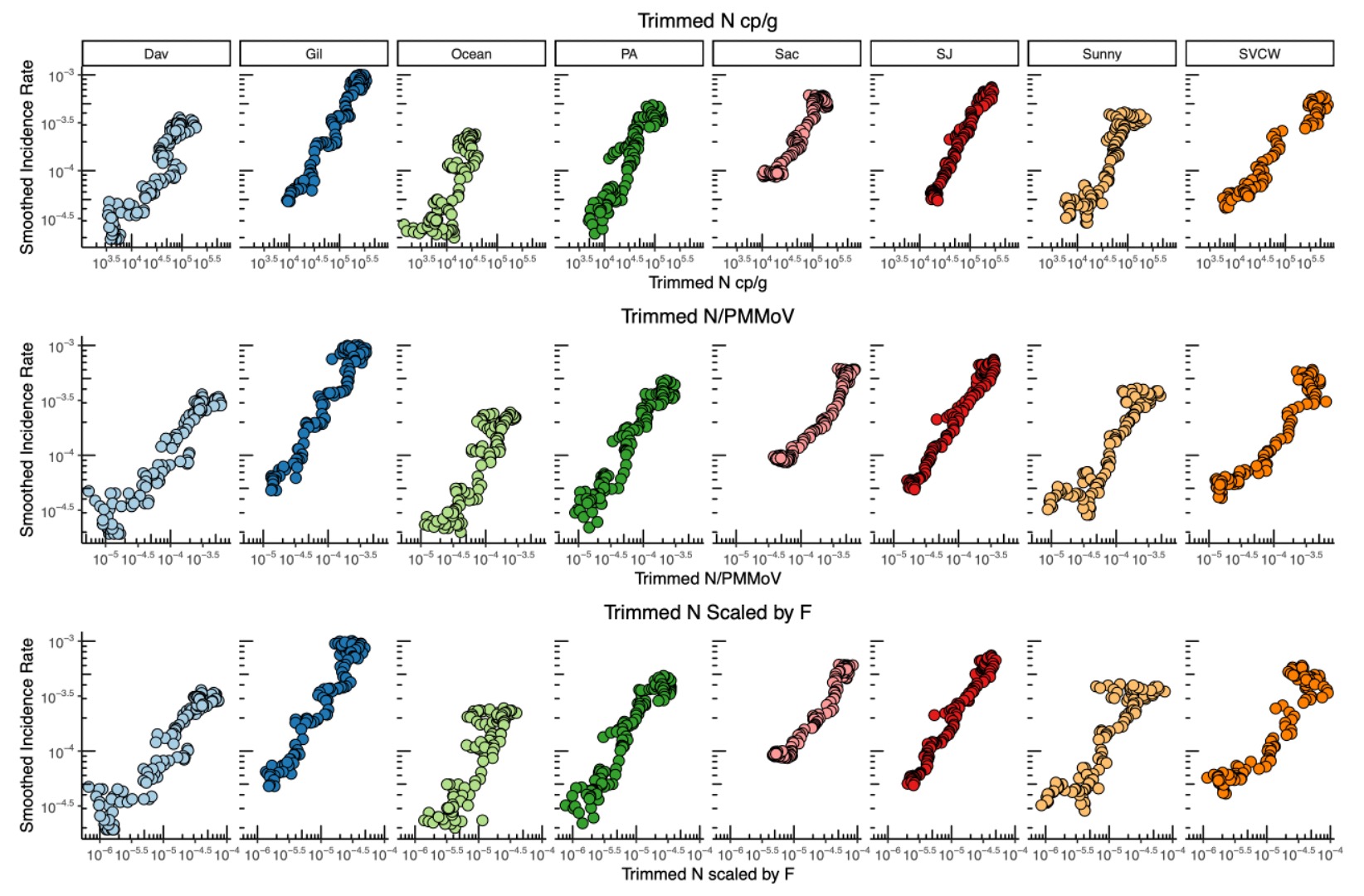

Supplement: FIG S6 [file msystems.00829-21-sf006.jpg]

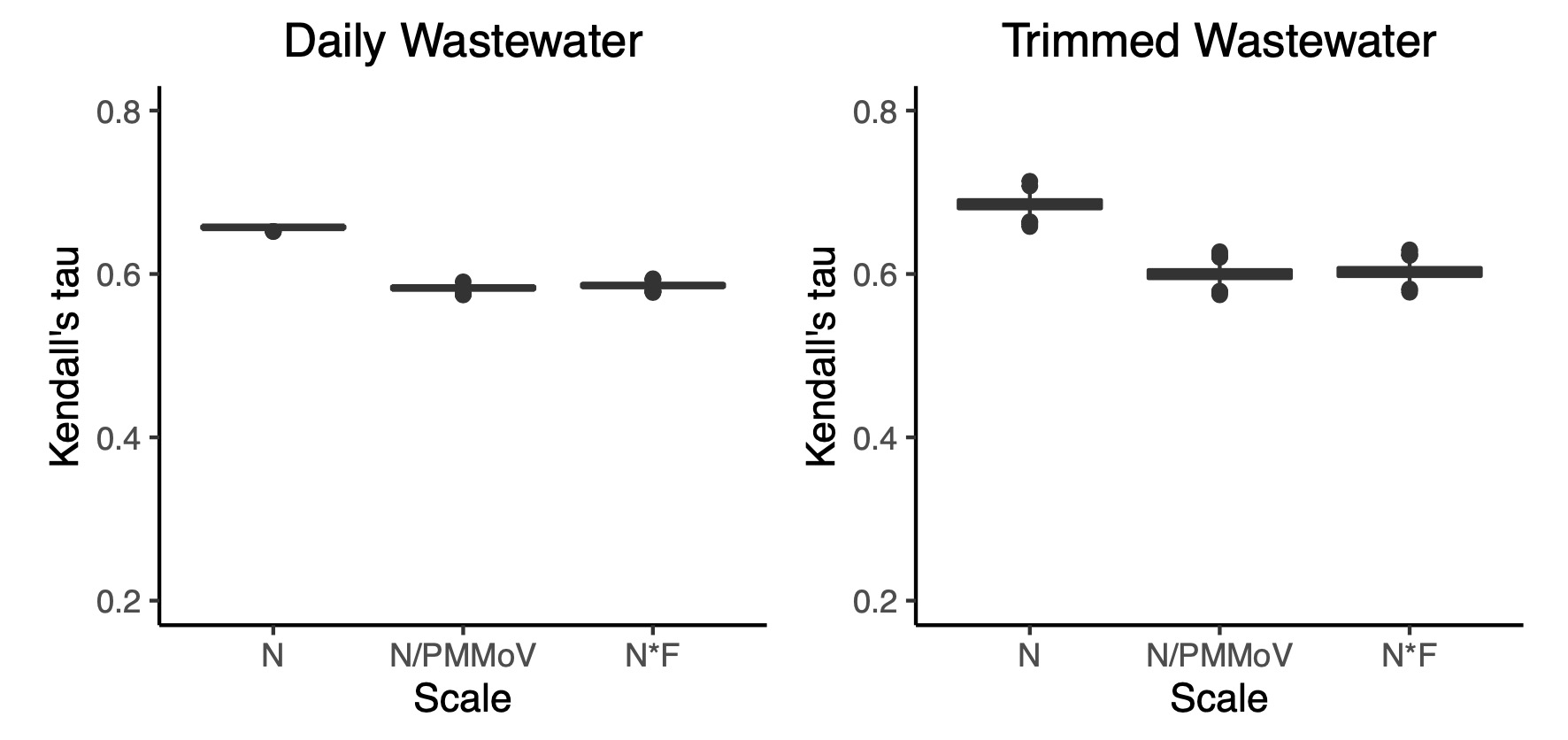

Supplement: FIG S7 [file msystems.00829-21-sf007.jpg]
