# Supplementary material for: High-Frequency, High-Throughput Quantification of SARS-CoV-2 RNA in Wastewater Settled Solids at Eight Publicly Owned Treatment Works in Northern California Shows Strong Association with COVID-19 Incidence
Source: mSystems. 2021 Sep 14;6(5):e00829-21. doi: 10.1128/mSystems.00829-21 (PMC8547422; doi:10.1128/mSystems.00829-21)
Supplement: TABLE S2 [file msystems.00829-21-st002.docx]

| POTW | TSS ave | TSS max | TSS min | TSS med | TSS stdev |
| --- | --- | --- | --- | --- | --- |
| Dav | 263 | 316 | 222 | 264 | 22 |
| Gil | 341 | 552 | 152 | 58 | 58 |
| Ocean | 346 | 4370 | 23 | 284 | 468 |
| PA | 218 | 264 | 160 | 220 | 23 |
| Sac | 281 | 420 | 130 | 280 | 48 |
| SJ | 306 | 384 | 246 | 305 | 25 |
| SVCW | 273 | 500 | 214 | 256 | 53 |
| Sunny | 228 | 376 | 164 | 216 | 56 |
