# Supplementary material for: High-Frequency, High-Throughput Quantification of SARS-CoV-2 RNA in Wastewater Settled Solids at Eight Publicly Owned Treatment Works in Northern California Shows Strong Association with COVID-19 Incidence
Source: mSystems. 2021 Sep 14;6(5):e00829-21. doi: 10.1128/mSystems.00829-21 (PMC8547422; doi:10.1128/mSystems.00829-21)
Supplement: TABLE S1 [file msystems.00829-21-st001.docx]

| **Target** | **Primer/Probe** | **Sequence** |
| --- | --- | --- |
| N Gene | Forward | CATTACGTTTGGTGGACCCT |
|  | Reverse | CCTTGCCATGTTGAGTGAGA |
|  | Probe | CGCGATCAAAACAACGTCGG (5’ FAM/ZEN/3’ IBFQ) |
| S Gene | Forward | CAGACTAATTCTCCTCGGCG |
|  | Reverse | TGCACCAAGTGACATAGTGT |
|  | Probe | AGCTAGTCAATCCATCATTGCCT (5’ HEX/ZEN/3’ IBFQ) |
| ORF1a | Forward | CAGAACTGGAACCACCTTGT |
|  | Reverse | TACAGTTGAATTGGCAGGCA |
|  | Probe | TGCCACAGTACGTCTACAAGC (5’ FAM or HEX/ZEN/3’ IBFQ) |
| BCoV | Forward | CTGGAAGTTGGTGGAGTT |
|  | Reverse | ATTATCGGCCTAACATACATC |
|  | Probe | CCTTCATATCTATACACATCAAGTTGTT (5’ FAM/ZEN/3’ IBFQ) |
| PMMoV | Forward | GAGTGGTTTGACCTTAACGTTTGA |
|  | Reverse | TTGTCGGTTGCAATGCAAGT |
|  | Probe | CCTACCGAAGCAAATG (5’ HEX/ZEN/3’ IBFQ) |
