# Supplementary material for: High-Frequency, High-Throughput Quantification of SARS-CoV-2 RNA in Wastewater Settled Solids at Eight Publicly Owned Treatment Works in Northern California Shows Strong Association with COVID-19 Incidence
Source: mSystems. 2021 Sep 14;6(5):e00829-21. doi: 10.1128/mSystems.00829-21 (PMC8547422; doi:10.1128/mSystems.00829-21)
Supplement: TEXT S1 [file msystems.00829-21-s0001.docx]

*### SET parameters ---------*

*# LHS*

*Kd <- 1000 # Kd for SARS ml/g*

*Kdp <- 100 # Kd for PMMoV ml/g is Kdp*

*lhs_scale = Kdp*(1+Kd*10^-6*tss_int)/(Kd*(1+Kdp*10^-6*tss_int)) # LHS model - scale to be applied to Cs/Cp*

*M = length(n_gc) # Set length of the data set*

*kk = 1000 # number of iterations to pick randomly*

*### DEFINE bootstrapped vectors for results for Kendall's tau ---------------*

*# define vectors for variable picks*

*N_gc_pick <- array(NA,c(M,kk))*

*N_pmmov_pick <- array(NA,c(M,kk))*

*pmmov_pick <- array(NA,c(M,kk))*

*LHS_N_pick <- array(NA,c(M,kk))*

*#for results of kendall's tau*

*N_gc_estimates <- array(NA,c(kk))*

*N_pmmov_estimates<-array(NA,c(kk))*

*LHS_N_estimates <- array(NA,c(kk))*

*N_gc_pvalues <- array(NA,c(kk))*

*N_pmmov_pvalues <-array(NA,c(kk))*

*LHS_N_pvalues <- array(NA,c(kk))*

*### generate PICKS from a uniform distribution for each wastewater variables --------*

*for(i in 1:M)*

*{*

*N_gc_pick[i,] = runif(kk, n_gc_lci[i], n_gc_uci[i])*

*pmmov_pick[i,] = runif(kk, pmmov_gc_lci[i], pmmov_gc_uci[i])*

*N_pmmov_pick[i,] = N_gc_pick[i,]/pmmov_pick[i,]*

*LHS_N_pick[i,] = N_pmmov_pick[i,]*lhs_scale[i]*

*}*

*### analysis of bootstrapped results for Kendall's tau ------------*

*for (i in 1:kk){*

*N_gc_result = cor.test(log10(N_gc_pick[,i]), log10(cases_prop_7ma), method = "kendall")*

*N_gc_estimates[i] = N_gc_result$estimate*

*N_gc_pvalues[i] = N_gc_result$p.value*

*#*

*N_pmmov_result <- cor.test(log10(N_pmmov_pick[,i]), log10(cases_prop_7ma), method = "kendall")*

*N_pmmov_estimates[i] = N_pmmov_result$estimate*

*N_pmmov_pvalues[i] = N_pmmov_result$p.value*

*#*

*LHS_N_result <- cor.test(log10(LHS_N_pick[,i]), log10(cases_prop_7ma), method = "kendall")*

*LHS_N_estimates[i] = LHS_N_result$estimate*

*LHS_N_pvalues[i] = LHS_N_result$p.value*

*}*
